# Carbapenem and amikacin resistance on a large conjugative *Acinetobacter baumannii* plasmid

**DOI:** 10.1093/jac/dku486

**Published:** 2014-11-27

**Authors:** Steven J. Nigro, Kathryn E. Holt, Derek Pickard, Ruth M. Hall

**Affiliations:** 1 School of Molecular Bioscience, The University of Sydney, Sydney, New South Wales, Australia; 2 Department of Biochemistry and Molecular Biology and Bio21 Molecular Science and Biotechnology Institute, The University of Melbourne, Parkville, Victoria, Australia; 3 Wellcome Trust Sanger Institute, Hinxton, Cambridge, UK

**Keywords:** *A. baumannii*, resistance plasmids, *aphA6*, *bla*_OXA-23_

Sir,

Worldwide, most multiply antibiotic-resistant (MAR), and particularly carbapenem-resistant, *Acinetobacter baumannii* causing infections belong to global clone 1 (GC1) or global clone 2 (GC2). Though there have been incidences where a different clonal group was predominantly responsible for an epidemic outbreak, MAR isolates that are not members of these clones have been observed to cause infections in hospitals but are not as common.^1^

Isolate D46, collected at Royal North Shore Hospital, Sydney in 2010, was shown to be resistant to ampicillin, imipenem, meropenem, ticarcillin/clavulanate, ceftazidime, cefotaxime, streptomycin, spectinomycin, sulfamethoxazole, tetracycline, trimethoprim, chloramphenicol, florfenicol, kanamycin, neomycin, gentamicin, amikacin, tobramycin, nalidixic acid and ciprofloxacin, making it extensively antibiotic resistant. D46 was previously shown to be ST110 (Oxford MLST) and to harbour the *aphA6* amikacin resistance gene within Tn*aphA6* and an *aadB* gene cassette (gentamicin, kanamycin and tobramycin resistance) in the small plasmid pRAY.^2^ An ISAba1 upstream of the chromosomal *ampC* confers resistance to third-generation cephalosporins, ceftazidime and cefotaxime.^3^

Here, we have further examined the causes of resistance. Using PCR as described previously,^4^ D46 was shown to contain the *strA*-*strB*, *sul2* and *tetA*(B) genes, responsible for streptomycin, sulfamethoxazole and tetracycline resistance, respectively. These genes were in the same configuration as in AbGRI1-2 (Tn*6167*),^4^ but D46 does not have an island in *comM*. Carbapenem and ticarcillin/clavulanate resistance was due to the *bla*_OXA-23_ carbapenemase gene, which was within Tn*2006*.

To better understand how Tn*2006* was acquired, conjugation was performed as described previously,^5,6^ using D46 as a donor and a rifampicin-resistant mutant of *A. baumannii* ATCC 17978 as a recipient. Transconjugants resistant to meropenem, imipenem and ticarcillin/clavulanate, indicative of *bla*_OXA-23_, and resistant to kanamycin, neomycin and amikacin, indicative of *aphA6*, were recovered. Hence, both Tn*2006* and Tn*aphA6* were located on a conjugative plasmid.

The whole genome sequence of D46 was determined using Illumina HiSeq and assembled as described previously.^5^ The draft genome comprised 115 contigs with an average read depth of 88.7× coverage and D46 was determined to be ST25 according to the Pasteur MLST scheme. Three plasmids were detected. The sequence of the smallest plasmid, pD46-1 (6078 bp), contained *aadB* and was almost identical to pRAY* (a single base difference).^2^ pD46-2 is an 8731 bp cryptic plasmid that was identical to p1ABTCDC0715 (GenBank accession number CP002523) from a GC2 isolate.

To determine whether D46 harboured Tn*2006* together with Tn*aphA6* on a plasmid related to pAb-G7-2^5^ and pD72-2,^7^ contigs that matched them were retrieved from the draft genome. Seven contigs were recovered and assembled using PCR.^5,7^ The amplicons were sequenced, finalizing the assembly. Plasmid pD46-3 has a 67 027 bp backbone sharing 99.99% identity (five single-base changes) with that of pD72-2 (Figure 1a). pD46-3 also harboured Tn*aphA6* in the same position as pD72-2 (Figure 1a). One end of two backbone contigs had inversely oriented fragments of ISAba1 and they were linked with *bla*_OXA-23_ in Tn*2006* using PCR (Figure 1a). The complete 74 916 bp sequence of pD46-3 was deposited in GenBank under accession number KM977710. As the backbone of pD46-3 was almost identical to that of pD72-2 and pAb-G7-2, pD46-3 also contained the complete set of transfer genes (Figure 1). This is the first report of a completely sequenced *A. baumannii* plasmid that has been demonstrated to simultaneously transfer resistance to carbapenems and aminoglycosides.

**Figure 1.**
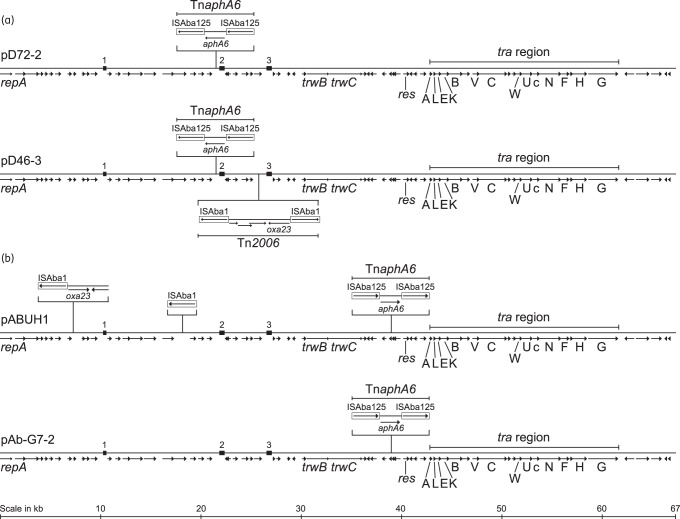
Comparison of *repAci6* plasmids pD72-2, pD46-3 (a), pABUH1 and pAb-G7-2 (b). The plasmid backbones, linearized and opened at *repA*, are represented by horizontal lines. The extent of ORFs and genes are shown as arrows beneath the lines, with names given where a function is known. The transfer region is indicated above each line, the *tra* genes are indicated with capital letters and *trbC* is shown as c. The three repeat regions are indicated by the numbered boxes. The structures of Tn*aphA6*, Tn*2006* and Tn*2008*-like are shown above or below the backbone, with IS represented as open boxes containing an arrow showing their orientation.

A recent genomic study of a GC2 *A. baumannii* collection from a US hospital reported the sequence of pABUH1 (GenBank accession number AYOH01000010), which the authors suggested could be transferable.^8^ It was described as a plasmid related to pACICU2, harbouring ISAba1 upstream of *aphA6* and having a copy of *bla*_OXA-23_ flanked by two copies of ISAba125 that is in the same position as ISAba125 in pACICU2.^8^ However, it was recently shown that pACICU2 is likely identical to pAb-G7-2 and in fact harbours Tn*aphA6* at this location.^9^ Our analysis of the sequence of pABUH1 revealed that it in fact carries Tn*aphA6* in this position (Figure 1b). Furthermore, ISAba1 is actually upstream of *bla*_OXA-23_, in a structure similar to Tn*2008* (GenBank accession number GQ861438) that is flanked by a 9 bp direct repeat. However, in pABUH1 the sequence adjacent to the ISAba1 was 1613 bp whereas it is only 1351 bp in Tn*2008*.

The backbones of the *repAci6* plasmids, pAb-G7-2, pD72-2, pD46-3, pABUH1 and pACICU2, are all very closely related, but they can be separated into two lineages based on the position of Tn*aphA6* (Figure 1). Representatives of the two lineages, pD46-3 and pABUH1, have each acquired the *bla*_OXA-23_ gene on separate occasions and a sixth *repAci6* plasmid, pA85-3, has also gained *bla*_OXA-23_ within AbaR4.^6^ The acquisition of *bla*_OXA-23_ in different structures and in separate events indicates that this group of related plasmids plays a vital role in the dissemination of this carbapenemase gene and could be one of the factors responsible for making it a worldwide problem. Hence, surveillance of this group of *A. baumannii* plasmids will be vital in curtailing the spread of carbapenem resistance in *A. baumannii*.

## Funding

This study was supported by grants from the School of Molecular Bioscience and the Wellcome Trust Sanger Institute. S. N. was supported by an Australian Postgraduate Award. K. E. H. was supported by NHMRC fellowship 628930.

## Transparency declarations

None to declare.
